# Management regulations for odontogenic keratocyst: a case report and review of the literature

**DOI:** 10.1186/s13256-024-04473-8

**Published:** 2024-04-05

**Authors:** Srishti Grover, Shreya Hegde, Roma Mascarenhas

**Affiliations:** Department of Conservative Dentistry and Endodontics, Manipal College of Dental Sciences Mangalore, Affiliated to Manipal Academy of Higher Education, Manipal, Karnataka India

**Keywords:** Biopsy, Developmental cyst, Odontogenic keratocyst, Periapical surgery, Endodontic therapy

## Abstract

**Background:**

Reconstruction of the entire dentition with odontogenic keratocyst is a very challenging quandary. Most cases of odontogenic keratocyst are often reported to be benign, resulting in severe occlusal discrepancies with the maxillary and mandibular dentition. Dental radiographs occasionally reveal an uncommon, locally aggressive developing cyst termed as odontogenic keratocyst, which is typically located in the posterior jaw. When this cyst occurs in the anterior region, it is often misdiagnosed with other periapical lesions due to its lack of response to pulp vitality tests.

**Case presentation:**

This clinical case scenario demarcates the endodontic management of a patient diagnosed with odontogenic keratocyst. A 37-year-old Indian male patient reported to the department with throbbing pain in the lower left posterior tooth requiring endodontic therapy. This patient also presented with odontogenic keratocyst in the anterior region of the jaw, for which he had undergone surgical rehabilitation. This case report highlights the clinical protocol for the endodontic therapy in patient diagnosed with ododntogenic keratocyst. Masticatory impairment was not visible after the follow-up period and the treatment outcome was successful.

**Conclusion:**

This case report details the presentation, characteristic radiographic findings, and endodontic management of a patient with an extremely rare condition of odontogenic keratocyst. The management involves multidisciplinary approach for the rehabilitation.

## Background

Endodontic therapy is required when periapical lesions with considerable alveolar bone damage and responsiveness to a tooth sensibility test are absent. Such endodontic lesions can have varied differential diagnosis, which can be grouped as periapical granuloma, radicular cyst, and lateral periodontal cyst, and it is common to misdiagnose odontogenic keratocysts (OKC) as different developmental cysts. OKC is a rare benign locally aggressive developmental cyst that accounts for about 19% of jaw cysts [1].

The term odontogenic keratocyst was originally described by Philipsen in 1956. He subsequently changed the name of this lesion to keratocystic odontogenic tumor (KCOT) [2]. According to the World Health Organization (WHO), it is a benign uni- or multicystic, intraosseous tumor of odontogenic origin (dental lamina and its remnants) with characteristic lining of parakeratinized stratified squamous epithelium and potential for aggressive and infiltrative behavior [3]. The posterior part and the lower ramus of the mandible are the most common site of occurrence, accounting for almost 50% [4].

Odontogenic keratocyst are usually camouflaged with larger cysts, such as ameloblastoma and dentigerous cysts [3], while the smaller ones resemble unilocular radiolucent lesions with corticated borders. These lesions are diagnosed in the second, third, and fourth decades of life and appear to be more common in male patients than female patients [3].

This unusual case report reported the presence of OKC in the anterior mandible that was successfully treated by surgical intervention and endodontic therapy of the posterior tooth on the left side of the jaw.

## Case report

**Fig. 1:**
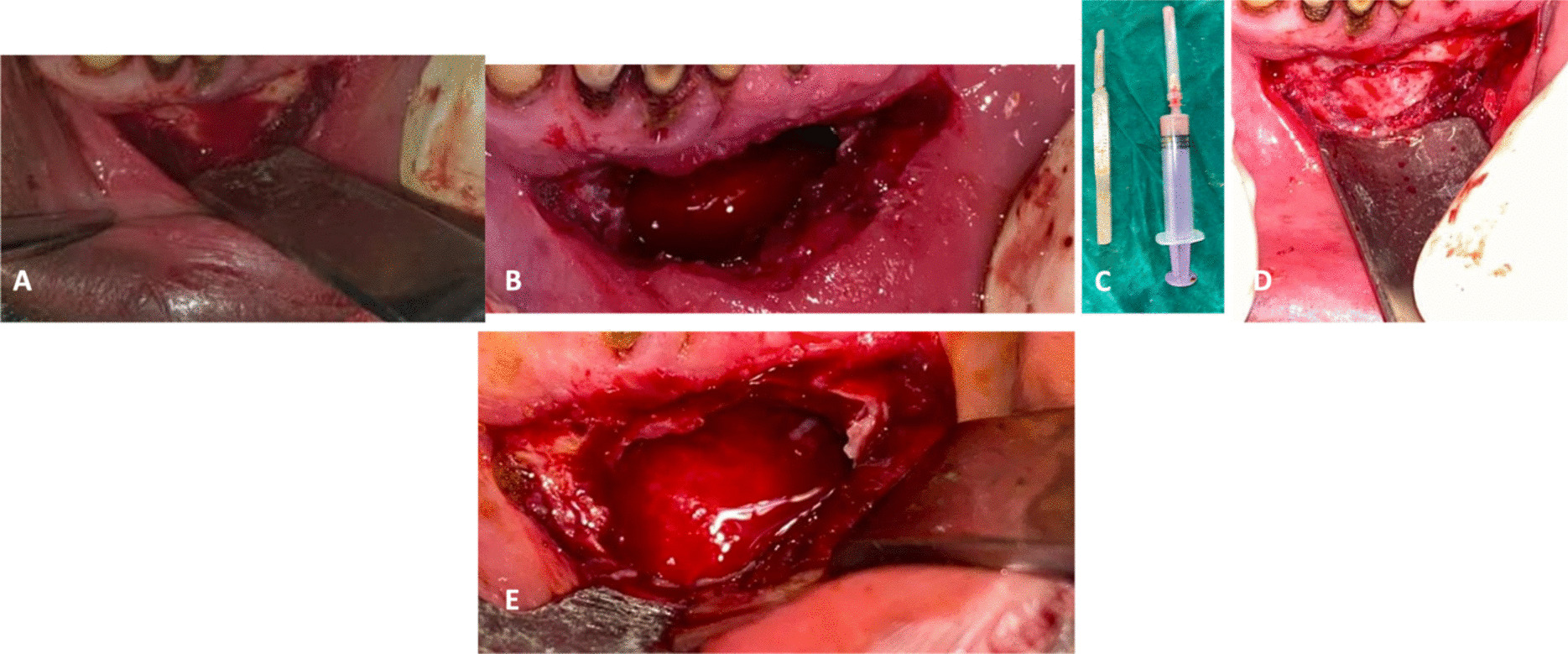
**A** Vestibular incision. **B** Exposure of the cystic site. **C** Aspirate containing cystic contents. **D** Cystic cavity depicting bare bone. **E** Cystic cavity showing cystic lining

**Fig. 2:**
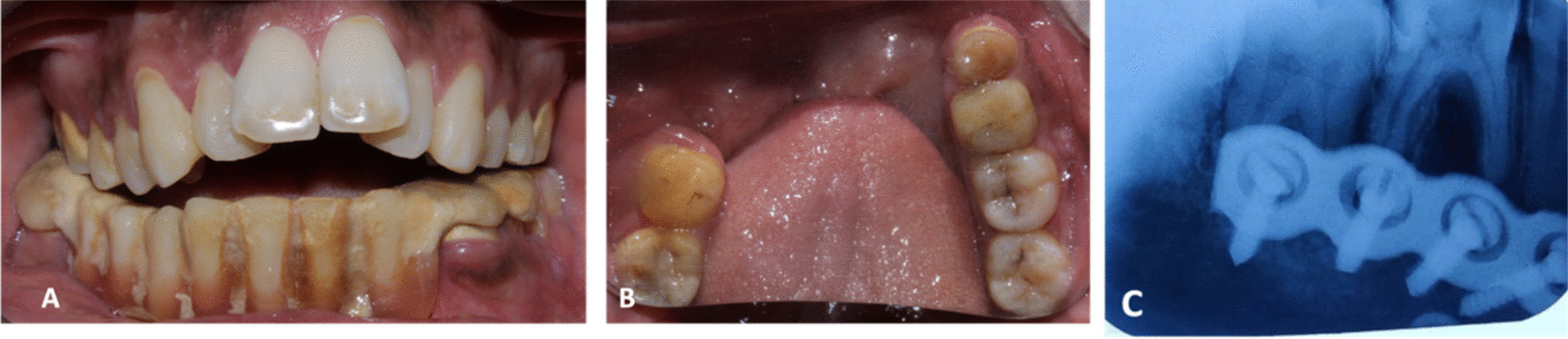
**A** Maximum intercuspation with the removable prosthesis. **B** Mandibular occlusal view with tooth of interest (36). **C** IOPAR depicting deep restoration

A 37-year-old Indian male patient reported to the department of Conservative Dentistry and Endodontics with the chief complaint of history of throbbing pain in the lower left back tooth region for 5 days. Patient presented with night pain, which did not subside even after taking analgesics. Family history and medical history was non-contributory. Patient gave a history of an odontogenic keratocystic (OKC) lesion in the anterior mandible that was managed by surgical intervention (Fig. 1) and followed by rehabilitation with a removable prosthesis for aesthetic purposes (Fig. 2A) Extra-oral examination disclosed normal mouth opening and no temporomandibular joint problems. The patient was healthy with no comorbidities.

On intraoral examination, a class II composite restoration was present with respect to tooth 36 (Fig. 2B). The tooth was tender on percussion. In addition, teeth 35 and 36 exhibited grade II mobility. Pulp sensibility testing gave an exaggerated lingering response. Radiographic examination revealed a well-defined radio opacity involving enamel, dentin, and pulp with periapical radiolucency (Fig. 2C), suggestive of deep restoration. Nonsurgical endodontic management was chosen as the treatment plan (Fig. 4).

**Fig. 3:**
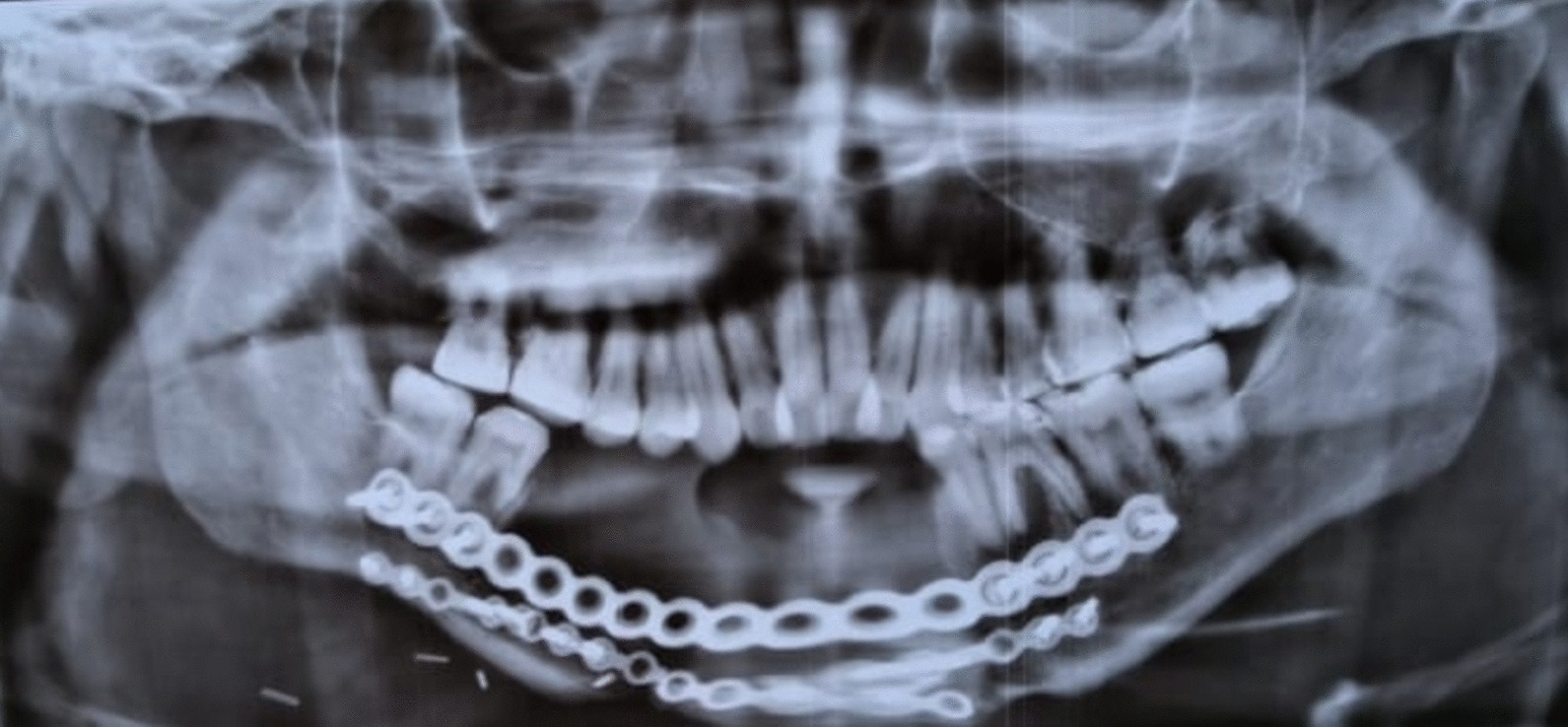
Orthopantomogram depicting segmental resection of anterior mandible reconstituted with reconstruction plates

Radiographic examination included full mouth orthopantomogram (OPG) and intraoral periapical radiograph (IOPA) of tooth 36. A panoramic radiograph (OPG) was taken after surgical intervention depicted resected OKC in the anterior mandible reconstituted with reconstruction plates (Fig. 3). Occlusion was assessed after obtaining diagnostic casts. A well-formulated treatment plan involving nonsurgical endodontic therapy was performed with respect to tooth 36. The mobility of the tooth (36) was reduced following endodontic therapy. The entire treatment plan was explained to the patient and informed consent was obtained. The initial phase of the treatment consisted of endodontic therapy. On the basis of the clinical diagnostic criteria for reduction in mobility, periodontic treatment was planned.

Treatment protocol: patient was asked to check on his diet to maintain oral hygiene. The patient was content with the anticipated outcome and the prognosis of the overall treatment plan was satisfactory. The subsequent rehabilitation plan was formulated.

**Fig. 4:**
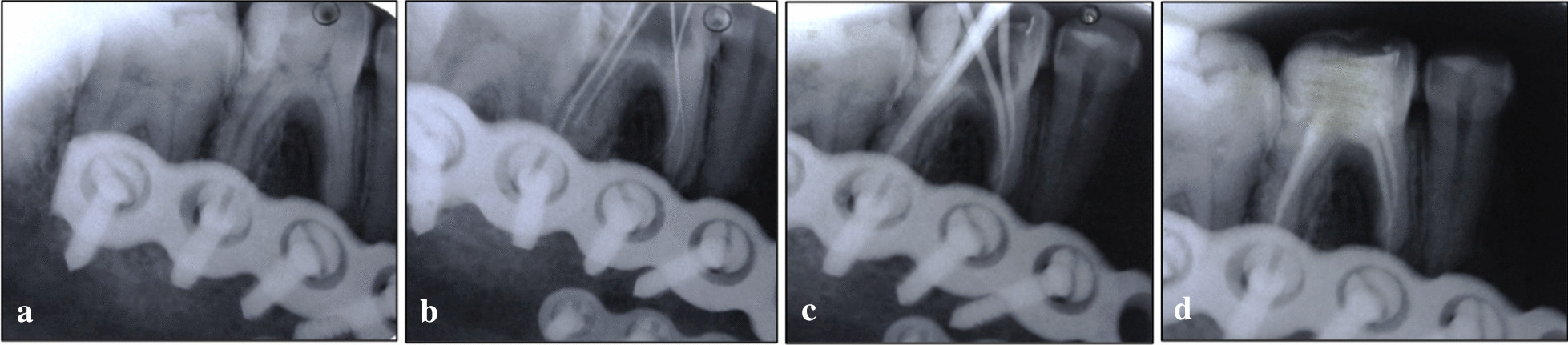
(**a**) Preoperative IOPAR. (**b**) Working length IOPAR. (**c**) Master cone IOPAR. (**d**) Obturation IOPAR with post-endodontic restoration

Definitive treatment plan:

1. Patient motivation for the treatment and maintenance of oral hygiene.
2. Endodontic treatments of tooth 36.
3. Periodic follow-ups at 6-month and 1-year intervals followed by augmentation of oral hygiene measures (Fig. 4).

Endodontic treatment was performed with respect to tooth 36 under rubber dam and local anesthesia, and 3% sodium hypochlorite (Vishal Dentocare Pvt Ltd, India) was used as irrigant. The working length was obtained using hand K file (Dentsply Inc, Maillefer, Dentsply India), Root ZX II Apex locator (Morita, Irvine, CA), and finally confirmed using intraoral periapical radiograph. Cleaning and shaping of all the canals were performed with hand K files and Protaper rotary files (Dentsply Inc, Maillefer, Dentsply India) under copious irrigation with 3% sodium hypochlorite and enlarged up to F2 file size. Two rounds of triple antibiotic paste (TAP) as intracanal medicine (3Mix-MP), which is a combination of metronidazole, minocycline, and ciprofloxacin in the ratio of 1:1:1, was applied to tooth 36 for a period of 1 week. Triple antibiotic paste has the capacity to sterilize the pulp space from all microbes and also has regeneration potential to induce root development. The access cavity was given temporary seal dressing (cotton pellet and intermediary restorative material, Cavit G, 3 M ESPE GmbH, Neuss, Germany). Finally, the obturation was completed using F2 protaper gutta percha cone (Dentsply, Maillefer, Dentsply India) with AH Plus sealer (Dentsply, Sirona, Dentsply India).

## Discussion and review of literature

The World Health Organization classifies odontogenic keratocyst as a developmental, noninflammatory odontogenic cyst arising from remains of dental lamina cells [5]. This developmental cyst occurs in the Ramus-third molar region of the mandible rather than the maxilla [6]. Odontogenic keratocyst has shown tendency for rapid progression and invasion of the adjoining tissues, including bone [7, 8].

Histologically, odontogenic keratocyst originates from the dental lamina and is composed of a cystic lined space consisting of desquamated keratin, uniform layer of parakeratinized squamous epithelium of 5–10 cell layers, and a basal layer of columnar or cuboidal cells with vertically oriented nuclei. The area next to the connective tissue is a flat surface acting as a storehouse for formation of basal layer with satellite cysts [9]. Compared with other cysts presenting with odontogenic origin, there is a faster rate of cellular division [10].

The literature has recommended several treatment options for OKC, from the most conservative, such as marsupialization or decompression, to the most radical, such as resection. The important factors influencing the treatment decision are the size and location of the lesion, its relationship to important adjacent structures, and whether it is a primary or recurrent condition [11]. This case represented an aggressive variant of OKC, which was confirmed in the biopsy report, hence a more radical approach, that is, enucleation with segmental resection of the anterior mandible was chosen. The mandibular anterior region was then rebuilt with reconstruction plates. The recurrence rates after resection have proven to be less, as stated in literature [12].

In this present case report, the patient presented with throbbing pain for a few weeks after surgical intervention, hence an orthograde endodontic treatment was planned for the patient. Intra oral Periapical radiograph (IOPAR) of tooth 36 showed periapical radiolucency involving enamel, dentin, and pulp, hence root canal therapy was performed with the placement of two rounds of triple antibiotic paste as intracanal medicaments [13].

Triple antibiotic paste (TAP) is considered the standard intracanal medicament and consists of metronidazole, minocycline, and ciprofloxacin. Literature has shown that TAP has a well-known track record in aiding the healing of periapical lesions [14]. Since the infected root canal flora contains a majority of obligate anaerobes, metronidazole is considered as the first-line drug in TAP. At high concentrations, metronidazole has failed to completely eradicate the microorganisms from the root canal, so a combination of drugs was suggested. Propylene glycol is considered the ideal vehicle for delivery of intracanal medicine [15].

The combination of triple antibiotic paste helped in almost 90% of the disinfection of the root canal. Metronidazole is a broad-spectrum antibiotic that is nitroimidazole derivative and highly efficacious against anaerobes and protozoa. Minocycline is a semisynthetic compound of tetracycline with similar broad-spectrum activity. Ciprofloxacin is a synthetic fluoroquinolone, which is highly effective against bacteria [16]. Hence, due to the above-mentioned advantages, TAP was chosen as the intracanal medicament for this case.

Pulpal death due to infections often leads to periapical lesions. However, odontogenic infections such as ododntogenic keratocyst often tend to simulate the signs and symptoms of pulpal necrosis, thereby making diagnosis and treatment planning difficult. Hence, it is of utmost importance for dental clinicians to include a multidisciplinary approach for such difficult cases. The patient was well satisfied with the treatment.

The entire treatment plan for a patient with odontogenic keratocyst (OKC) should be aimed at rehabilitating the entire stomatognathic system. The final treatment modality to date still revolves around strengthening the masticatory system [10].

Odontogenic keratocyst (OKC) constitutes heterogeneous category disorders, which are classified as odontogenic inflammatory cysts (radicular, periapical, and collateral inflammatory cysts) and odontogenic and non-odontogenic cysts. This developmental cyst is considered rare and unique due to its high recurrence rate and local aggressive activity. Odontogenic keratocyst is also peculiar owing to its histological characteristics. OKCs evolve from dental lamina and are composed of a cystic space with desquamated keratin, 5–10 uniform cell layers of parakeratinized squamous epithelium, and a definite basal, columnar, or cuboidal cell layer with vertically oriented nuclei. The juncture with abutting connective tissue is flat and has the capacity for the sprouting of basal layer and the development of new satellite cysts. The mitotic activity with OKC is greater than other odontogenic cysts [12].

Due to the histological characteristic, aggressive nature of the cyst, a significant proportion of disorders are associated with the mutation or deactivation of tumor suppressor gene, also called protein patched homolog (PTCH) gene [14].

Restoration of oral condition of patients with OKC requires a lot of patience, skill, and accuracy, as well as extensive cooperation with other dental sectors. Multidisciplinary approach is mandatory for the rehabilitation of odontogenic keratocyst cases.

## Conclusion

Management of patients with odontogenic keratocyst poses a constant challenge to the dentist. Treatment strategies for ododntogenic keratocyst requires multidisciplinary approach to achieve successful results. Endodontic treatment for such cases should be handled with specialist supervision to prevent the possibility of reinfection and contamination.

## Data Availability

Not applicable.

## Abbreviations

OKC: Odontogenic keratocyst
OPG: Orthopantomogram
IOPA: Intraoral periapical radiograph
EDTA: Ethylenediaminetetraacetic acid
